# Neonatal birth asphyxia and associated factors among newborns delivered and admitted to NICU in selected public hospitals, under Addis Ababa City Administration Health Bureau, Addis Ababa, Ethiopia, A cross-sectional study

**DOI:** 10.1186/s13052-024-01761-3

**Published:** 2024-09-18

**Authors:** Dawit Tarko, Tesfu Zewdu, Shewamene Tesfaye, Abel Gerezihear, Azeb Haile

**Affiliations:** 1 Research Department, Gandhi Memorial Hospital, Addis Ababa, Ethiopia; 2 Department of Nursing, School of Health Sciences, Ambo University Woliso Campus, Woliso, Ethiopia; 3 Department of Nursing, College of Health Sciences, Assosa University, Assosa, Ethiopia; 4 Public Health Emergency management Department, Zewditu Memorial Hospital, Addis Ababa, Ethiopia

**Keywords:** Birth, Asphyxia, Neonate, Addis Ababa

## Abstract

**Background:**

In developing countries birth asphyxia is a major cause of neonatal morbidity and mortality. Despite the implementation of various strategies and interventions to combat neonatal mortality rates, birth asphyxia remains the main public health concern in Ethiopia. Moreover, limited studies have been conducted, especially in the study area and there are no multicenter analyses available to generate evidence for action. Therefore, this study aimed to assess the burden and associated factors of birth asphyxia among newborns in the selected public hospitals of the Addis Ababa City Administration Health Bureau.

**Methods:**

Three hundred forty-three mother-child pairs who used delivery services and gave birth in the selected public hospitals were included in the study, and institution based cross sectional study design was employed. A systematic random sampling technique was used to select the study participants. A pretested, structured interviewer administered questionnaire was used to collect the data. The physician’s/health care professionals diagnosis of an Apgar score less than 7 within the first five minutes of life led to the confirmation of the diagnosis of birth asphyxia. SPSS version 24 was used for analysis after the data were exported from Epi Info version 7.2. Multivariate logistic regression analysis included variables which had P-values less than 0.25 in the bivariable logistic regression analysis. The study findings were expressed using adjusted odds ratio with a 95% confidence interval, and P-value less than 0.05 was used to declare the statistical significance.

**Results:**

The magnitude of birth asphyxia was found to be 17.1% [95% CI; (13.2–21.5)] at the first 5 min. In the multivariable logistic regression analysis cord accident [AOR = 6.24: 95% CI; (1.24–31.32)], prolonged duration of labor [AOR = 2.49: 95% CI; (1.93–10.89)], and meconium-stained amniotic fluid [AOR = 3.33: 95% CI; (1.73–6.41)] were the predictors of birth asphyxia.

**Conclusions:**

The findings of this research indicate that birth asphyxia is a prevalent neonatal problem at the study area. Therefore, the Addis Ababa Health Bureau must prioritize integrated mitigation interventions targeting high-risk pregnancies to achieve national and international commitment to sustainable changes in newborn health.

## Background

Neonatal birth asphyxia is defined as the failure to establish breathing following delivery, or a condition in which the baby does not receive enough oxygen before, during, or immediately after birth. Neonatal morbidity and mortality have been increased dramatically worldwide due to neonatal hypoxia. The main cause of perinatal asphyxia is complications during childbirth [1, 2].

The American College of Obstetricians and Gynecologists and the American Academy of Pediatrics clearly stated the criteria to diagnose neonatal asphyxia(consider neonatal asphyxia if the following conditions are fulfilled: umbilical cord arterial pH < 7; Apgar score of 0–3 for longer than five minutes; neurological manifestations like seizures, hypercapnia, metabolic acidosis, and hypoxic-ischemic encephalopathy) [3, 4]. According to the World Health Organization (WHO) report, birth asphyxia accounts for an estimated 900,000 deaths each year [5]. An estimated 2.5 million newborns die worldwide annually accounting for approximately 47% of all under-5 mortality, and 54% of all under-five deaths occur during the neonatal period among African babies [6, 7].

Most newborn deaths (around 75%) occur in the first week of life, with about one million newborns dying in the first 24 h. Among these newborns, the leading causes or risk factors of death are premature birth, birth complications (newborn asphyxia/trauma), prolonged labor, premature rupture of membrane, high blood pressure, cord accidents, neonatal infections, and congenital anomalies, which together account for nearly 4 out of 10 deaths of children under 5 years of age [8, 9]. The Ethiopian neonatal mortality rate (NMR) is 30/1000 live births [10], and 31.6% of neonatal mortality is attributed to birth asphyxia [11].

Studies conducted in Ethiopia showed that the prevalence of birth asphyxia at Dire Dawa was 3.1% [12], at Debre Tabor General Hospital was 28.35% [13], and the pooled prevalence of birth asphyxia in the country was 19.3% [14]. Despite the decrease in child mortality rate worldwide, there are striking differences between regions and countries [15]. For example, neonatal mortality in sub-Saharan Africa was ten times higher than in high-income countries [16].

For example, that issue of neonatal morbidity and mortality, although less marked, is present also in high-income countries, with significant disparities among different geographic regions even in the same country, as observed in Italy. The Pediatric culture and the neonatological care, which grew in the last decades, contributed to the decrease of such rates, but for some vulnerable populations or patients at higher risk (e.g. FGR newborns) the strategies and efforts to improve perinatal care still need to be implemented by healthcare professionals and institutions [17–19].

Asphyxia which occurs during childbirth is associated with reduced access to skilled care during pregnancy, delivery and the postpartum period. Women who received an ongoing care and health information guided by a midwife and regulated according to the international standards were 16% less likely to lose their baby and 24% less likely to have a premature birth [5, 20].

Birth asphyxia can be prevented by implementing different strategies such as increasing antenatal care coverage, skilled delivery and assistance [21].

Neonatal resuscitation guidelines endorsed by the world health organization (WHO) and the American Academy of Pediatrics are used as a standard practice for improving outcomes in asphyxiated newborns [5]. There is also an established treatment guideline for birth asphyxia in Ethiopia and around 19% of newborns suffer from this problem (19%) [14, 22].

In order to provide adequate and rapid resuscitation measures to asphyxiating newborns, it is important to understand the burden and factors affecting birth asphyxia. Moreover, limited studies have been performed, especially in the study area and there are no multicenter studies conducted to generate evidence for action. Therefore, this study aimed to assess the burden and associated factors of birth asphyxia among newborns admitted to the NICU in the selected public hospitals of the Addis Ababa City Administration Health Bureau.

## Methods

### Study area and period

The study was conducted in four public hospitals, administered by the Addis Ababa City Administration health bureau. The population of the city was 7.8236 Million (2019 –Estimation) [23]. The Addis Ababa City Administration has twelve referral hospitals, three primary hospitals, 106 public health centers and 722 private medium and higher clinics, and 48 private hospitals. These health facilities have their own different departments like Gyn/Obs, surgery, pediatrics, ophthalmology, and internal medicine. The selected hospitals have a pioneer maternal and neonatal health services [24]. The study was conducted from March to April 2024.

### Study design

An institution based cross sectional study design was used.

### Population

#### Source population

The source population was represented by all neonates, with their mothers, delivered and admitted to NICU in Addis Ababa city public hospitals.

#### Study population

All neonates, with their mothers, delivered and admitted to NICU in the selected public hospitals under Addis Ababa City Administration Health Bureau.

### Eligibility criteria

#### Inclusion criteria

- All newborns, with their mothers, delivered and admitted to NICU in the selected public hospitals.
- Newborns who were born after 28 weeks of gestation.

#### Exclusion criteria

- Incomplete documentation (no maternal or fetal measurement parameters).
- Mothers who took general analgesia and seriously ill.
- Neonates with congenital heart defects.

### Sample size determination

The sample size was calculated for both first and second objectives and the sample size calculated for the first objective was larger than the sample size calculated for the second objective. By using a single-population proportion formula with assumptions; the proportion (P) = 28.35% [13], Z-the standard normal distribution value at 95% confidence level of Zα/2 = 1.96, 5% of absolute precision, and 10% non-response rate, sample size was: -

$$\:n=\frac{\left({Z\frac{\alpha\:}{2}\:)}^{2}*P\right(1-P)}{{d}^{2}}$$

$${{{{\left( {1.96} \right)}^2}*(0.2835*(1 - 0.2835))} \over {\left( {0.0025} \right)}} = 312.134 \sim 312$$

By taking 10% non-response rate, 10% * 312 = 31, the final sample size was 343.

### Sampling procedure

The study was carried out in four selected public hospitals namely Gandhi memorial hospital, Zewditu memorial hospital, Abebech Gobena memorial hospital, and Tirunesh Beijing hospital. The final sample size was distributed for the above-mentioned public hospitals proportionately based on the number of last month neonates delivered and admitted to NICU in each selected hospital (by considering the last one month’s prior report). A systematic random sampling technique was employed to approach the study subjects. The sampling fraction (Kth) was calculated, and then every 2 intervals the study subjects were approached.

### Study variables

#### Dependent variable

- Neonatal birth asphyxia.

#### Independent variables

- Socio-demographic characteristics:
  - Age, residence, marital status, educational status, income level.
- Antepartum related characteristics:
  - Parity, Antenatal care (ANC), number of ANC visits, previous delivery history, medical complications, bad obstetric history, years of birth spacing.
- Intrapartum related characteristics:
  - Time of membrane rupture, type and duration of labor, mode of delivery, place of delivery, fetal presentation, amniotic fluid, cord accident, time of delivery.
- Neonatal related characteristics:
  - Sex, gestational age, weight of the baby.
- Maternal health behavioral related factors:
  - Alcohol drinking, cigarette smoking, chat chewing.

### Operational definition

Birth asphyxia: Neonates born in the studied hospitals and diagnosed as asphyxia by an attendant professional with an Apgar score of < 7 within the first 5 min. The definition of neonatal asphyxia of AAP is mainly used to diagnose sever asphyxia. We used the above operational definition (Apgar score of < 7 within the first 5 min) because it enabled us to include all mild, moderate and severely asphyxiated newborns.

Prolonged labor: labor exceeding 12 h in primigravida or 8 h in multipara mothers after the latent phase of the first stage of labor.

Premature rupture of membranes (PROM)): rupture of the membrane of the amniotic sac and chorion that occur > 1 h before the onset of labor.

### Data collection tools and procedures

A structured questionnaire was developed first in English language, after reviewing pertinent literature that have been published previously [12, 13, 22, 25]. A pretested, structured interviewer- administered questionnaire was used to collect data on maternal socio-demographic profile. Data related to antepartum (such as parity, antepartum hemorrhage, and antenatal visits), intrapartum (such as fetal presentation, mode of delivery, meconium-stained amniotic fluid, and premature rupture of membrane), and neonatal factors (such as gestational age, birth weight, and sex) and also maternal health behaviors, were extracted using a pretested structured checklist from the medical records of the neonates and their mothers. First five-minutes Apgar score was collected by the data collectors for every newborn. Data collection process was conducted by trained bachelor of science degree holder midwives, nurses and supervised by master of science degree holders.

### Data quality management

Three days training was given to the data collectors and supervisors on the purpose of the study, the methods and tools of data collection, and how to reduce the possibility of bias. The supervisors and the investigators were followed and coordinated the field work throughout the data collection period; every completed data collection form was checked for consistency and completeness by investigators and the supervisors. Pretest was done on 5% of the same source population at the non-selected health institution, which was not included in the final sample size and then based on the findings of the pretest the questionnaire was modified as necessary. Data were double-entered into Epi-info version 7.2 to ensure their quality.

### Data processing and analysis

The collected data were coded and entered into Epi info version 7.2, and exported to SPSS version 24 for analysis. Bivariable and multivariable logistic regression analyses were done. Tables, figures, pie chart and text were used to present the results of the analyzed data. Independent variables with P-value < 0.25 in the bivariable logistic regression analysis were considered for multivariable logistic regression analysis, and P-value < 0.05 was used as cut off point to declare the statistical significance. Multicollinearity was checked. Finally, model fitness was checked. The Hosmer Lemeshow test of goodness of fit, which considers good fit at P-value *≥* 0.05, was used to determine the final model’s goodness of fit.

## Results

### Socio-demographic characteristics

A total of 340 study participants responded to the questionnaire, with response rate of 99%. The majority of the study participants (144, 42.4%) belonged to the age group 25–29 years, with mean and standard deviation of 27.6 ± 4.502 respectively. With regard to the place of residence and marital status, more than half of them 302(88.8%) lived in urban area and three hundred one (88.5%) of them were married respectively (Table 1).

**Table 1:** Socio-demographic characteristics of the study subjects, Addis Ababa, Ethiopia, 2024 (*n* = 340)

Variable	Category	Frequency	Percent
Age of the mothers	15–19	10	2.9
	20–24	74	21.8
	25–29	144	42.4
	30–34	87	25.6
	*≥* 35	25	7.4
Place of residence	Urban	302	88.8
	Rural	38	11.2
Marital status	Single	29	8.5
	Married	301	88.5
	Divorced	8	2.4
	widowed	2	0.6
Educational status	No formal education	29	8.5
	Primary education	81	23.8
	Secondary education	110	32.4
	Certificate and above	120	35.3
Average monthly income (Ethiopian Birr)	*≤* 5350	121	35.6
	5351–7800	82	24.1
	7801–10,900	99	29.1
	*≥* 10,901	38	11.2

### Occupational status

**Fig. 1:**
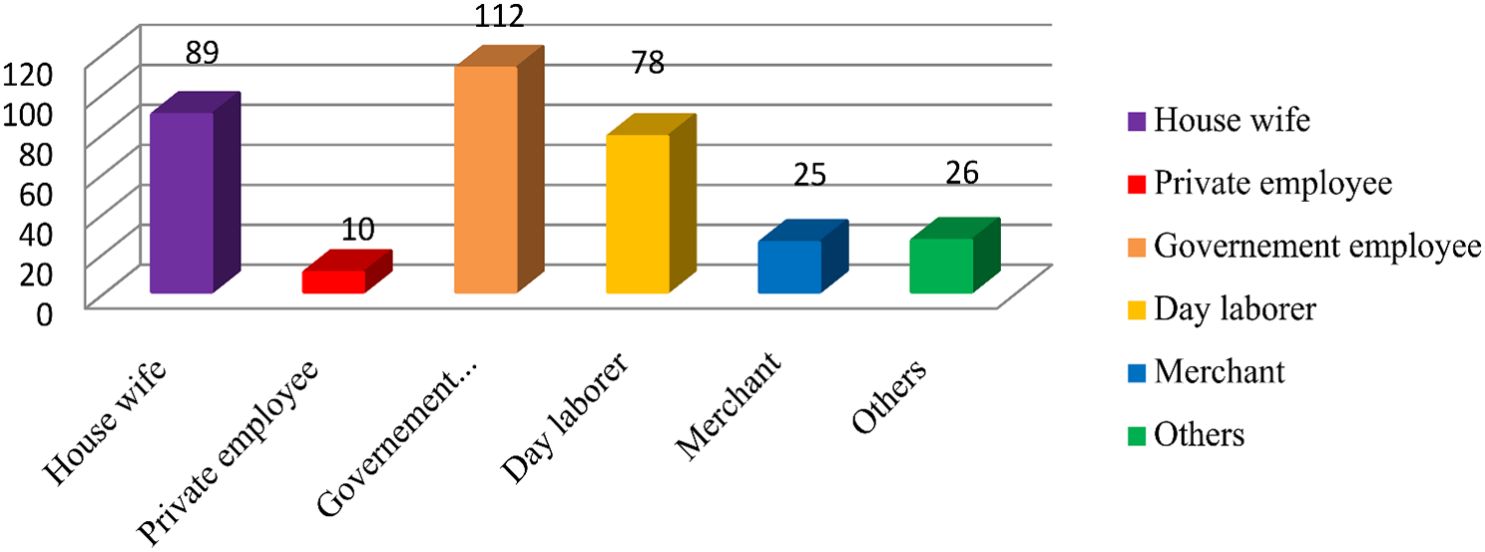
Occupational status of the study subjects in the selected public hospitals, Addis Ababa, Ethiopia, 2024 (*n* = 340)

Out of the total study participants, 112 (32.9%) and 89 (26.2%) of them were government employee and house wife respectively (Fig. 1).

### Antepartum related characteristics

In this study, 326 (95.9%) of the study subjects had ANC follow up, and among these, 297 (91.1%) had more than four ANC visits. From those study participants who had ANC follow up, 186 (57.1%) of them started their first ANC visit during their second trimester of pregnancy. From the total study subjects, 185 (54.4%) of them were multiparous, and 41 (22.2%) of them had a history of adverse pregnancy outcomes; 104 (30.6%) had obstetric complications during their current pregnancy (Table 2).

**Table 2:** Antepartum related characteristics of the study participants in the selected public hospitals, Addis Ababa, Ethiopia, 2024 (*n* = 340)

Variable	Category	Frequency	Percent
ANC follow-up	Yes	326	95.9
	No	14	4.1
Number of ANC follow-up (*n* = 326)	Only once	4	1.2
	2–3 times	25	7.7
	*≥* 4 times	297	91.1
Gestational age (GA) at first visit of ANC (*n* = 326)	1st trimester	114	35
	2nd trimester	186	57.1
	3rd trimester	26	8.0
VDRL result	Reactive	9	2.6
	Non-reactive	327	96.2
	Unknown	4	1.2
Parity	Primipara	155	45.6
	Multipara	185	54.4
Pregnancy interval in years (*n* = 185)	< 1	11	5.9
	1–2	63	34.1
	*≥* 3	111	60
Previous pregnancy adverse outcome (*n* = 185)	Yes	41	22.2
	No	144	77.8
Current pregnancy obstetric complication	Yes	104	30.6
	No	236	69.4
PROM (*n* = 340)	Yes	125	36.8
	No	215	63.2

### Intrapartum related characteristics

Of the total study participants, 273 (80.3%) gave birth spontaneously. More than half of the study subjects, 316 (92.9%) had vertex fetal presentation. From all the study subjects, 21 (6.2%) faced obstructed labor, 83 (24.4%) experienced meconium-stained amniotic fluid during delivery, and 186 (54.7%) gave birth at night (Table 3).

**Table 3:** Intrapartum related characteristics of the study subject in the selected public hospitals, Addis Ababa, Ethiopia 2024 (*n* = 340)

Variable	Category	Frequency	Percent
Types of labor	Spontaneous	273	80.3
	Induced	67	19.7
Duration of labor	Normal labor	228	67.1
	Prolonged labor	112	32.9
Mode of delivery	Spontaneous vaginal delivery	144	42.4
	Instrumental	39	11.5
	Cesarean section delivery	157	46.1
Fetal presentation	Cephalic	316	92.9
	Non –cephalic	24	7.1
head-obstructed labor	No	319	93.8
	Yes	21	6.2
Color of amniotic fluid	Stained	83	24.4
	Clear	257	75.6
Grade of amniotic fluid	Grade-I	17	20.5
	Grade-II	42	50.6
	Grade-III	24	28.9
Cord accident	No	330	97.1
	Yes	10	2.9
Time of delivery	Day	154	45.3
	Night	186	54.7
Substance use	Chat chewing	6	22.2
	Cigarette smoking	3	11.1
	Alcohol drinking	18	66.7

### Neonatal related characteristics

From the total delivered and admitted neonates, 159 (46.8%) were females, and 232 (68.2%) had a normal birth weight. Based on the category of gestational age of the newborns, 268 (78.8%) of them were born at term. Of almost all newborns, 316 (92.9%) were singleton (Table 4).

**Table 4:** Neonatal related characteristics of the study subjects in the selected public hospitals, Addis Ababa, Ethiopia, 2024 (*n* = 340)

Variable	Category	Frequency	Percent
Sex	Male	181	53.2
	Female	159	46.8
Weight	Low birth weight	98	28.8
	Normal birth weight	232	68.2
	Macrosomic baby	10	2.9
Gestational age	Preterm	72	21.2
	Term	268	78.8
Weight for age	AGA	302	88.8
	SGA	32	9.4
	LGA	6	1.8
Birth outcome	Single	316	92.9
	Twin	22	6.5
	Triplet	2	0.6
Apgar score at the first 5 min	Low	2	0.6
	Intermediate	56	16.5
	Normal	282	82.9
Stage of perinatal asphyxia	Stage-I	19	32.8
	Stage-II	29	50
	Stage -III	10	17.2
Admission diagnosis at NICU	Hypothermia	61	18
	RDS	175	51.6
	EONS	88	26
	PNA	58	17.1
	NHB	52	15.2
	Others	15	4.4

Others (Meningitis, TTN, Cord bleeding, IUGR), AGA = appropriate for gestational age, SGA = small for gestational age, LGA = large for gestational age, RDS = Respiratory distress syndrome, EONS = Early onset neonatal sepsis, PNA = Perinatal asphyxia, NHB = Neonatal hyperbilirubinemia

### Magnitude of neonatal birth asphyxia

**Fig. 2:**
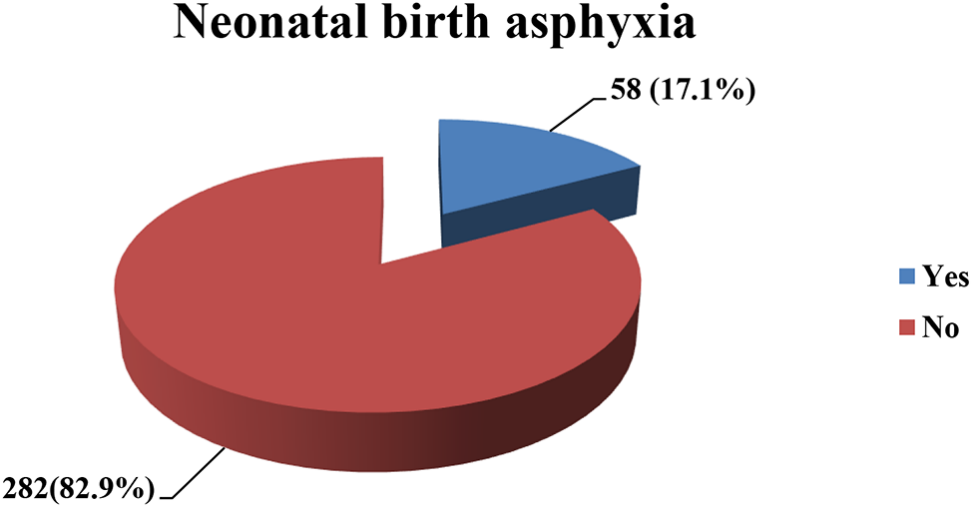
Magnitude of neonatal birth asphyxia in the selected public hospitals, Addis Ababa, Ethiopia, 2024 (*n* = 340)

The magnitude of birth asphyxia was found to be 17.1% [95% CI; (13.2–21.5)] (Fig. 2).

### Factors associated with neonatal birth asphyxia

In the bivariable logistic regression analysis mothers who were experienced an obstructed labor, premature rupture of membrane, meconium-stained amniotic fluid, cord accident, night time delivery, and those labored for an extended period of time, non-cephalic fetal presentation, and mode of delivery were factors affecting neonatal birth asphyxia at P-value less than 0.25.

On multivariable logistic regression analysis, meconium-stained amniotic fluid, cord accident and prolonged duration of labor were the independent predictors of birth asphyxia at P-value < 0.05 (Table 5).

**Table 5:** Factors associated with neonatal birth asphyxia in the selected public hospitals, Addis Ababa, Ethiopia, 2024

Variables	Category	Birth asphyxia		COR 95% CI	AOR 95% CI	*P*-value
		Yes	No			
Time of delivery	Day	20	134	1	1	
	Night	38	148	1.72[0.95–3.10]	1.62[0.89–3.34]	0.11
Color of amniotic fluid	Stained	30	53	4.63[2.55–8.40]	3.33[1.73–6.41]	0.000*
	Clear	28	229	1	1	
Duration of labor	Normal	28	200	1	1	
	Prolonged	30	82	2.61[1.47–4.65]	2.49[1.93–10.89]	0.04*
Cord accident	Yes	6	4	8.02[2.19–29.41]	6.24[1.24–31.32]	0.03*
	No	52	278	1	1	
Obstructed labor	yes	8	13	3.31[1.31–8.40]	1.17[0.34–4.04]	0.81
	No	50	269	1	1	
Fetal presentation	Cephalic	50	266	1	1	
	Non -cephalic	8	16	2.66[1.08–6.55]	2.14[0.70–6.54]	0.19
Mode of delivery	Spontaneous vaginal delivery	23	121	1	1	
	Instrumental	12	27	2.34[1.04–5.27]	1.90[0.73–4.90]	0.19
	Cesarean section delivery	23	134	0.90[0.48–1.69]	0.65[0.32–1.31]	0.23
Presence of PROM	Yes	26	99	1.50[0.85–2.66]	1.14[0.58–2.26]	0.71
	No	32	183	1	1	

Note *represents *P* < 0.05, PROM = Premature rupture of membranes

## Discussion

Birth asphyxia can cause death or lifelong complications in newborns. Therefore, information on the burden and underlying factors of asphyxia is needed to develop contextual interventions, that are critical to reduce overall neonatal morbidity and mortality. In this study, the prevalence of neonatal birth asphyxia among neonates delivered and admitted to the NICU in the study area was found to be 17.1% (95% CI (13.2–21.5)). This study finding is in line with studies conducted in East and Central Africa (15.9%) [26], in Ethiopia (19.3%) [14], in Hawassa (17.9%) [27], in Jimma (18%) [28], in Harari and Dire Dawa (20.8%) [29]. This overlapping might be due to the similarity of the study population and the institutions where the study was conducted.

Also, the rates of asphyxia found in our study are higher than those observed in Uganda (5.3%) [30], in Kenya (5.1%) [31], in North Central Ethiopia (11.11%) [32], in Northeastern Ethiopia (13.1%) [33]. This discrepancy might be due to the differences in health care coverage and maternal problems, and where some mothers are referred from other health care facilities to the selected hospitals of this study for further investigation and care for their severe maternal complications.

In addition, the result of this study is lower than studies done at Dilla (32.8%) [22], at Siltie Zone Worabe south central Ethiopia (41.2%) [34], at Wolayita Zone southern Ethiopia (26.4%) [35], North east Amhara Ethiopia (22.6%) [36], at Debre Tabor north central Ethiopia (28.35%) [13]. This difference may be due to the quality of care that the pregnant mothers received during their ANC visits in the study area, the educational status of the mothers, and their health-seeking behavior or also to the differences in the socio-cultural characteristics of the subjects of the present study.

The findings of this study showed that newborns who encountered cord accident were 6.24 times more (AOR = 6.24; 95% CI: ( [1.24–31.32]) likely to develop birth asphyxia as compared to their counterparts. This finding is supported by studies conducted in Northern Tigray, and Wolayita Sodo [37, 38]. The possible explanation could be linked with problems with the umbilical cord, which may lead to its compression, interrupting the normal exchange of blood, nutrients, and oxygen, or to the compression of the arteries.

Meconium-stained amniotic fluid was also another identified factor associated with neonatal birth asphyxia in this study. The likelihood of birth asphyxia among neonates who faced stained amniotic fluid were 3.33 times higher (AOR = 3.33; 95% CI: ( [1.73–6.41]) as compared to their counterparts. This finding is consistent with studies conducted in Rwanda [39], in Kenya [40], in Northeast Amhara, Debre Tabor general hospital, in South Central Ethiopia, at Wolayita Sodo, Addis Ababa Ethiopia [13, 34, 36, 37, 41]. The possible reason of such association might be correlated with the presence of inflammation/infection conditions.

Lastly, neonates born through prolonged labor were 2.49 times [AOR = 2.49; 95% CI: (1.93–10.89]) more likely to be asphyxiated as compared to those who born through normal duration of labor. This finding is similar with studies done in different parts of Ethiopia [14, 37]. The possible reason might be due to the lack of oxygen following prolonged labor activity while the fetus is in the birth canal, leading to neonatal asphyxia. The main limitations of this study were: the study address only the association between outcome of interest and predictor variables not the effect of the predictors on the outcome variable and the study did not include neonates delivered at home and other health centers.

## Conclusion and recommendation

Neonatal asphyxia is a common problem in Addis Ababa, and factors significantly associated with birth asphyxia were cord accident, prolonged duration of labor and meconium-stained amniotic fluid. Therefore, health care providers, especially those working in labor departments must pay more attention to complicated births, prevention and early intervention to prevent birth asphyxia. Governmental and non-governmental agencies should be called upon to commit to the promotion and education of advanced obstetric and perinatal services and their use in the community.

## Data Availability

The data used during this study are available from the corresponding author on reasonable request.

## Abbreviations

AOR: Adjusted Odd Ratio
COR: Crude Odd Ratio
NICU: Neonatal Intensive Care Unit
SPSS: Statistical Package for social science
WHO: World Health Organization
